# Advanced Design and Manufacture of Mechanoactive Materials Inspired by Skin, Bones, and Skin-on-Bones

**DOI:** 10.3389/fbioe.2020.00845

**Published:** 2020-08-25

**Authors:** Melissa Louise Knothe Tate

**Affiliations:** Inaugural Paul Trainor Chair of Biomedical Engineering, Director MechBio Team, Graduate School of Biomedical Engineering, Faculty of Engineering, University of New South Wales, Sydney, NSW, Australia

**Keywords:** Microscopy-Aided Design And ManufacturE, mechanoactive materials, composite, biomimicry, additive manufacturing, smart materials, emergent properties, similitude

## Abstract

Life is mechanobiological. Natural living materials exhibit remarkable, emergent and smart properties under mechanical loading. Such materials are classified as *mechanoactive*, in contrast to electroactive polymers and materials that exhibit advanced properties when subjected to electrical stimulation. Cutting edge, multiscale imaging technologies have proven enabling for the elucidation of molecular to meso-scale structure and function of natural mechanoactive materials. Using Microscopy-Aided Design And ManufacturE, (MADAME) this perspective article describes mechanoactive properties of natural materials including skin-on-bones (*periosteum*) and bone itself. In so doing, it demonstrates the principle to emulate natural smart properties using recursive logic, the basis of many computer algorithms, and to design and manufacture mechanoactive materials and products using advanced manufacturing methods that also incorporate principles of recursive logic. In sum, the MADAME approach translates physically the computer science paradigm of recursion by implementing Jacquard textile methods, which themselves form a historical basis for computing machines, together with additive manufacturing methods including multidimensional printing, stereolithography, laser sintering, etc. These integrated methods provide a foundation and translational pathway for scaled-up manufacture of disruptive mechanoactive materials that will find use in fields as varied as medicine, safety, transport and sports, for internal (implants) and external (wearables) applications.

## Introduction

Nature abounds with stimuli-responsive, so-called *smart* materials. Examples of such materials, at the macro- to meso-length scale, include skin, bones, and skin-on-bones (*periosteum*) in the animal kingdom, and eucalyptus tree bark, cambium, and wood in the plant kingdom. Connective tissues comprising skin and other soft animal tissues exhibit remarkable mechanical strength, functional barrier properties to prevent moisture loss to the environment, while also “waterproofing” the internal organs, as well as self-healing and -sensing (e.g., pressure sensing) capacities (Knothe Tate et al., 2009; Tee et al., 2012). Vascular tissues of trees generate hydraulic pressure pulses when they bend in the wind (Lopez et al., 2014) and bone exhibits flow directing properties under mechanical loading, emerging from different calibers of interconnected vascular, pericellular, and matrix porosities (Knothe Tate et al., 2009). While top-down approaches to designing and manufacturing such smart materials have met with little success, bottom-up approaches using paradigms of “cellular manufacture” have been met with great success (Knothe Tate, 2011a,b; Tee et al., 2012; Knothe Tate, 2017b; Sidler et al., 2018).

**FIGURE 1:**
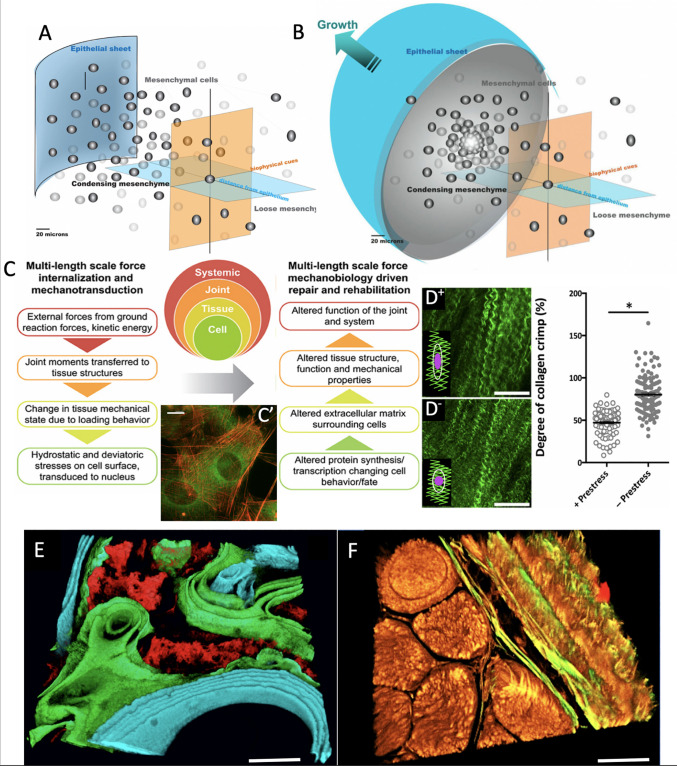
Imaging based studies to elucidate multi-time and length scale properties of tissues, using bone and skin-on-bones (periosteum) as examples. Transitions between Epithelial Sheets **(A)** and Mesenchyme **(B)** occur throughout life, providing tissue geometries for functional boundaries and tissue structures (Knothe Tate et al., 2008). **(C)** At every length scale, mechanical forces are transduced to and sensed by cells, which serve as actuators to build structural proteins that confer toughness, elasticity and turgor to tissue constructs (Ng et al., 2017b). **(C’)** After exposure to subtle cues such as 0.8 Pa fluid flow, cells buttress themselves by adapting their cytoskeleton (actin in red) and manufacturing structural proteins that are secreted to the extracellular matrix (collagen autofluorescing in green), thus modulating the cell’s own local environment (McBride et al., 2008). Scalebar: 5 μm. **(D^+^)** Once collagens are fully assembled in the extracellular matrix, they resemble curled or crimped structures which provide a scaffold for adherence of quiescent stem cells. Here, in the matrix of the periosteum, the tissue is tacked on to bony surfaces through an abundance of Sharpey’s fibers which help the tissue maintain a state of prestress. **(D^–^)** Once these fibers are cut or disturbed through periosteal lifting surgery or trauma, the prestress in the tissue relaxes; (see graph at right) and the collagen crimp increases significantly when prestress is removed, resulting in rounding of cells, a hypothetical trigger for stem cell egression to sites of injury (Yu et al., 2017). In this way, the “brainless” cells and the matrix interact as smart sensors and transducers and actuators. **(E)** When administered at known points in time, mineral chelating fluorochromes mark areas of mineralization which can be decoded to show temporal dynamics of mineralization under specific excitation and emission frequencies of light (Knothe Tate, 2011b). Scalebar: 50 μm. **(F)** Mineral nucleates around collagens. The collagens (yellow) and elastins (green) making up the tissue demonstrate the textile structure of tissues (muscle fascicles in lower left quadrant with bone weave in upper right quadrant, and periosteum in between) (Ng et al., 2017c). Together with the organic and inorganic extracellular matrix components, the tissue represents a complex composite structure (Knothe Tate, 2016, 2018, 2019). Scalebar: 50 μm. *Figure adapted and compiled from previously published figures, with permission.*

**FIGURE 2:**
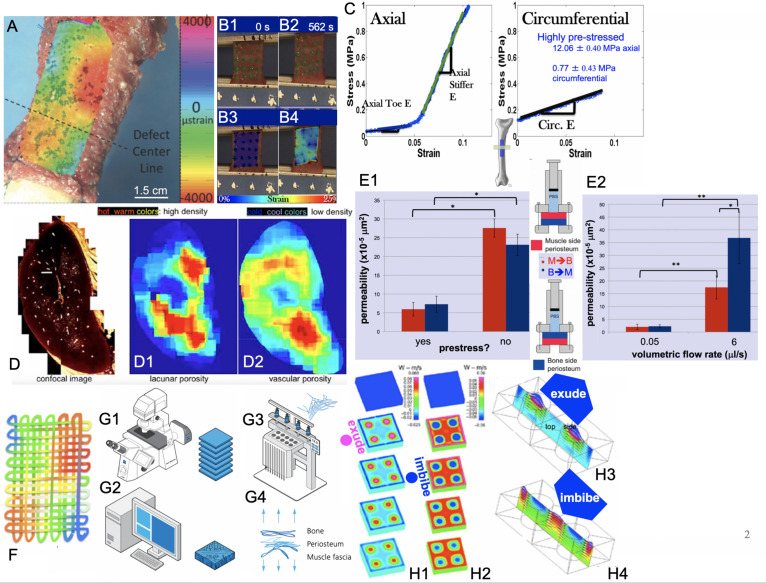
Smart properties of mechanoactive materials, including skin-on-bones (periosteum, **A,B,C,E**) and bone itself **(D)** and MADAME approaches to emulate those properties **(F,G1–G4,H1–H4)**. **(A)** Micron-resolution strain mapping of periosteum *in situ* under stance-shift loading demonstrates surprising heterogeneity (McBride et al., 2011a). **(B1–B4)** Similar heterogeneity is observed in strains during tensile testing of periosteum samples taken in the Axial and Circumferential directions **(C)** of the anterior (front) femur, which exhibit significant anisotropy (McBride et al., 2011b,c). (C – Axial) Specimens taken along the length of the femur exhibit strain stiffening while those taken along the circumference (C – Circumferential) of the femur exhibit linear elasticity. Removal of the samples through cutting of the Sharpey’s fibers and sample edges results in grossly observable shrinking of the specimens, which is significantly greater along the length of the long bone (Axial) compared to its circumference (McBride et al., 2011c). Based on shrinkage and measured moduli of elasticity, prestress of the tissue *in situ* can be calculated (Evans et al., 2013a). **(D)** Bone shows several calibers of porosity, including predominantly porosity around osteocytes (lacunar) and blood vessels (vascular). When the density of lacunar and vascular porosities are depicted spatially and as a heat map with warmer colors depicting higher density, the different patterns of lacunar **(D1)** and vascular **(D2)** densities are striking. Experimentally based computational models reveal counterintuitive flow effects when one explicitly accounts for these different spatial patterns of porosity densities, e.g., exudation of fluid under tension and imbibement of fluid under compression, which is contrary to daily experience with wet kitchen sponges (Reynolds, 1883; Knothe Tate et al., 2009). **(E1,E2)** Permeability studies on periosteum show a strong increase in permeability when prestress is removed from the tissue through cutting of Sharpey’s fibers. In addition, permeability depends strongly on volumetric flow rate and direction of flow, where flow in the bone to muscle direction increases significantly more than that in the opposite direction with increase in volumetric flow rate; *p* < 0.05 defines significant differences, * within, and ** between groups (Evans et al., 2013c). **(F)** Mechanical patterns resulting from intrinsic tissue weaves of e.g., elastin and collagen can be recreated recursively to create textiles emulating strain patterns observed in tissues. In this case, color patterns represent patterns of strain or fiber stiffness. Conceptually, “unraveling” of the tissue results in a singular solution with a fiber of varying stiffness along its length (Knothe Tate, 2016, 2017a,b, 2018; Ng et al., 2017c). An infinite number of solutions can be achieved through algorithms encoding patterns of stiffness, where microscopy acquired images **(G1)** provide a basis for infinite digital patterns and virtual prototypes **(G2)** that can be turned into physical prototypes using computer-controlled textile and knit systems, alone and/or in combination with other advanced manufacturing modalities such as multidimensional printing, laser sintering, etc. As a whole, the process is referred to as Microscopy-Aided Design And ManufacturE (Knothe Tate, 2016, 2018, 2019; Ng et al., 2017c). *Figure adapted and compiled from previously published figures, with permission.*

Remarkably, the “brainless” cells that manufacture all of the aforementioned smart materials, themselves form living sensors, actuators and transducers at the nano- to micron length scale (Knothe Tate and Niederer, 1998; Mishra and Knothe Tate, 2003; Knothe Tate and Anderson, 2013; Knothe Tate et al., 2016b; Ng et al., 2019, 2020). The advent of imaging across length- and time-scales has enabled not only unprecedented elucidation of the mechanisms underpinning cells’ and natural materials’ smart properties (Figure 1), but also the design and manufacture of new materials emulating nature’s own (Figure 2; Knothe Tate, 2017b). MADAME refers to a computer-aided additive manufacturing platform that incorporates Multi-D printing and/or computer-controlled weaving to create novel, bio-inspired materials and products (Steck et al., 2000; Knothe Tate, 2003; Sidler et al., 2018; Ng et al., 2019). The state of the art imaging capacity enables observation of live cells in their native tissue habitats. Design thinking processes empower engineers to empathize with their cells, imagining and feeling what they experience and envisioning their responses. In empathizing with their cells, engineers may be better equipped to prototype mechanoactive materials and architectures as cells do, from raw materials that they themselves produce and adapting their own structure and function, and ultimately their own niche to survive (Knothe Tate et al., 2016b).

This *perspective article* expands upon and integrates these topics to establish a foundation for advanced design and manufacture of mechanoactive materials that will be relevant for fields of use as varied as the medical and transport sectors, as well as for external and internal applications.

## Cells as Sensors, Actuators, and Transducers

Throughout nature, cells are the master designers, manufacturers and builders of tissue architectures underpinning e.g., trees and their population forests in the plant kingdom, as well as organs and organismal systems in the animal world (Knothe Tate et al., 2010). Cells literally differentiate themselves in their degree of *differentiation*, a biological term for structural and functional specialization. From undifferentiated stem cells, to terminally differentiated cells as diverse as dendritic bone and brain cells^+^, the structure and architecture of the cell encodes structural and functional memories of the cell’s life experience. Repeated activities reinforce structural connections (e.g., of the cytoskeleton, cell-matrix and cell-cell connections, etc.) which are stabilized over time, resulting in an adaptive functional memory of cell’s experiences throughout life (Knothe Tate and Fath, 2016; Knothe Tate et al., 2016b; Knothe Tate, 2017a). Cells’ dynamic stability and agility are a function of the cells’ structural stability as well as adaptive capacity, in context of cellular time scales for e.g., division, motility, structural protein expression and secretion, etc. The cytoskeleton is one repository of cellular information and the totality of cell structures at any given moment in time serve as an integrated sensor for the health state of the tissue and/or organ at that point in time and geographic location (Knothe Tate and Fath, 2016; Knothe Tate et al., 2016b).

Since cells sense their local environment and transduce biophysical (mechanical, electrical, osmotic, etc.) information to the nucleus, where gene up- and down regulation leads to stabilization of the cell and/or the cell’s environment over time, the cell itself is also an actuator of structural and architectural change as well as new local and global equilibria (Knothe Tate et al., 2016b; Figure 1C). By sensing and transducing information from its environment to the nucleus, where the basic building block proteins of tissues are created and secreted vectorially (with a magnitude/concentration and direction) (Knothe Tate et al., 2011a; Moore et al., 2014, 2016) to the extracellular matrix, the cell actively influences force balances at all relevant interfaces – between cells, cells and the matrix, and even between the cytoplasm and the nucleus. Cells are indeed smart in a materials science context, if not in a brain science context.

## Tissues Cells Weave and Their Smart Properties

Natural materials such as animal and plant tissues are natural composites comprising resilient collagens (animals) and celluloses (plants) that confer toughness, elastin (animals) and elastin-like polypeptides (plants) that impart elasticity, and proteoglycans that bind water and give turgidity. Cells “spin and weave” components of tissues *in situ* (Knothe Tate, 2011b, 2017b; Ng et al., 2017c) – the remarkable stimuli-responsive (smart) and adaptive properties of tissues emerge macroscopically from the directional, cellular secretion of nanoscopic extracellular matrix proteins as well as their anisotropic multicellular assembly and dis-assembly (polymerization and depolymerization). While in the future, the capacity to guide cells to manufacture smart materials in a controlled way may become possible, the current focus of this author’s R&D program is on emulating nature’s paradigms, either through scaled-up solutions based on similitude theory (Anderson et al., 2008; Knothe Tate, 2017b) or recursive logic based approaches (Steck et al., 2000; Knothe Tate, 2017b; Ng et al., 2017c). An overview is provided below, with specific examples inspired by skin, bone, and skin-on-bones in the following sections.

### Geometry

The success of Lego building blocks, with their universal interlocking mechanism, is their flexibility to build structures only limited by the creative capacity of the human brain. Nature’s counterpart could be the cells’ capacity to organize into sheets (*epithelial sheet*, Figure 1A) and three-dimensional, globular structures (*mesenchyme*, Figure 1B) that inter-convert throughout life. Indeed, all-natural tissue architectures are based on permutations of these sheets and globular structures, and development depends on the capacity of cells to revert between the two geometric design modalities, processes respectively referred to as epithelial-mesenchymal transition (EMT) and mesenchymal-epithelial transition (MET) (Morali et al., 2004; Knothe Tate et al., 2008, 2011a, 2016b; Evans et al., 2013c).

Tissues deriving developmentally from the *epithelial sheets* provide essential barrier functions to tissues at interfaces between different environments, e.g., skin provides a barrier between richly hydrated tissues of the body (∼85% water by volume) and the outside environment and the skin-on-bones *periosteum* covers all non-collagenous bone surfaces of the body, separating the *interior milieu* from the outside environment of bone, e.g., muscle compartments, fat, etc. (Figures 1F, 2A,B). Tight junctions between cells of epithelial sheets underpin this barrier property with their interpenetrating, zipper-like closures between cell membranes (Knothe Tate, 2003; Morali et al., 2004; Knothe Tate et al., 2008; Evans et al., 2013c).

In contrast, more globular *mesenchyme* consists of cells and extracellular matrix, joined by a variety of cell-cell and cell matrix junctions that act as molecular rivets and more distributed Velcro-like attachments that each, respectively, attach to proteins of the cell’s own skeleton (the cytoskeleton). *Mesenchymal condensation*, an event that occurs 11.5 days after fertilization in the mouse, initiates the formation of the musculoskeletal system. During this event, cells and their nascent tissues begin to specialize and rapidly scale-up cell number through cell division (*proliferation*) and formation of tissue templates through production of extracellular matrix (Morali et al., 2004; Knothe Tate et al., 2008; Evans et al., 2013c). Throughout the process, new geometries and architectures are stabilized by cell-cell and cell-matrix junctions.

Throughout prenatal development as well as during postnatal healing, which repeats prenatal development processes, cells interconvert between epithelial and mesenchymal states (EMTs and EMTs, as *per* above), enabling the formation of complex geometries, tissue architectures, and organ systems (Figures 1A,B; Morali et al., 2004; Putra et al., 2020). The process is so ubiquitous that the formation of deleterious tissues such as cancerous tumors and metastasis of circulating tumor cells follow analogous paradigms (Jolly et al., 2017). In many ways, this interconversion of geometries provides a most basic archetype for tissue architectures.

### Mechanical Modulation of Polarity to Anisotropy

Just as mechanical forces modulate EMTs and METs as tissue architectures evolve, they also direct self assembly of cell layers at the earliest stages of tissue organization. Forces including cell adhesion and cell tension modulate tissue patterning of tissues and influence tissue phenotype from the time of fertilization until after birth and throughout life. As mechanisms by which forces inherent to life on Earth translate to self-assembly of multicellular structures, including tissues and organs, this knowledge provides direct inspiration for bottom-up design, engineering and manufacturing of mechanoactive materials and devices (Figures 1C, 2F,G; Knothe Tate et al., 2008). Also of great relevance, from the earliest stages of cell polarization to formation of materials exhibiting anisotropy, mechanical interactions and concentration gradients, at the interface of the cell and its environment, guide the transcription and secretion of structural proteins, the building blocks of tissues, in anisotropic structures that translate to anisotropic (in terms of mechanical properties) or directional functions and properties (Evans et al., 2013b).

## Mechanoactive Materials

*Electroactive polymers* and *materials* change shape or size under electrical stimulation, while *mechanoactive* fibers and materials exhibit stimuli-responsive (*smart*) properties under mechanical loading (Figure 2). Given that life itself is mechanobiological, it is surprising that mechanoactive materials are less recognized and researched than electroactive materials. For example, if one compares search engine results for *mechanoactive* and *electroactive materials*, in PubMed, the former garner 14 compared to 4000 hits and, via google, 11,000 versus 2,200,000 respective results. The plethora of electroactive materials and applications parallels industry and manufacturing activities around the development of electronics and electronic components, and their scale down in size over time. In the future, one would expect increased publication and patent activity around scaling down of advanced materials’ and device manufacturing to include smart properties that emerge from smaller to larger length scales.

Interestingly, while top-down engineering approaches have led to elucidation of multiscale structure-function relationships in a variety of natural materials and tissue types (Spalazzi et al., 2008), bottom-up approaches appear more conducive to the elucidation, engineering and manufacture of *emergent* mechanoactive properties (Knothe Tate, 2011b; Evans et al., 2013c; Knothe Tate et al., 2016a). Emergence refers to properties or patterns that arise from the putting together of simpler elements which in themselves do not exhibit similar properties or patterns. The concept is further explained by example below.

### Periosteum, the Skin-on-Bones

Periosteum, a hyper-elastic soft tissue sleeve, envelops all non-articular (excluding the joint surfaces covered in cartilage) bony surfaces of the body, like a “skin-on-bones.” As an inherently “smart” material, soft periosteum imparts hard bones with added failure strength under high impact loads (Evans et al., 2013a,b,c; Knothe Tate et al., 2016c). Recent studies of periosteum’s mechanoactive properties reveal cellular and structural mechanisms underpinning its myriad smart properties, including anisotropic stiffness (modulus of elasticity) (McBride et al., 2011c), direction-dependent intrinsic prestress (McBride et al., 2011c; Yu et al., 2017) and permeability (Evans et al., 2013c), and a stem cell-triggering molecular weave that switches state upon release of prestress (Yu et al., 2017).

First, high definition television (HDTV) lenses were used to map in high resolution (submicron scale), four-dimensional (xyzt) strains in periosteum during stance shift loading of the sheep femur (McBride et al., 2011a,b). The rationale was to understand the local environment of stem cells that reside in a quiescent state in the periosteum until injury occurs. The working hypothesis was that the cells sense local strains in the periosteum which trigger them to egress from the periosteum in injury-inducing loading scenarios. Interestingly, 4D-imaging of strains in the periosteum revealed surprising heterogeneity in space and time (Figure 2A and Supplementary Video 1). Furthermore, areas of new tissue genesis via stem cells egressing from the periosteum correlate to areas with the largest shift in baseline strain rather than absolute strain magnitudes (McBride et al., 2011a).

Further paired imaging-mechanics studies on sections of periosteum from the longitudinal (axial, along the long bone) and/or circumferential, anterior (front) aspect of the sheep femur demonstrated a stark anisotropy in mechanical properties of the periosteum (McBride et al., 2011c). When loaded in tension, axial oriented sections (Figures 2B,C-Axial), exhibit significant strain stiffening above 0.05, while circumferentially oriented sections (Figures 2B,C-Circumferential) exhibit linear elastic behavior (McBride et al., 2011c).

Periosteum is attached to all bony surfaces via a plethora of collagen connections called *Sharpey’s fibers*, which “Velcro” the soft tissue to the hard bony surface (Ng et al., 2017c; Yu et al., 2017). When injured, or during orthopedic surgeries involving periosteal lifting, the Sharpey’s fibers become severed. In parallel with the mechanics studies described above, shrinkage of periosteum upon release from the underlying bone surface was quantified. Similar to mechanical stiffness, tissue shrinkage exhibited direction-dependence, whereby upon release, the tissue sections shrank significantly more in the axial compared to the circumferential direction. These relative shrinkages were used to calculate an intrinsic prestress in the tissue (Figure 2C), revealing that the tissue is highly prestressed in the axial direction (12.06 ± 0.40 MPa) and much less so in the circumferential direction (0.77 ± 0.43 MPa) (McBride et al., 2011a; Evans et al., 2013a).

Follow on high resolution microscope imaging studies revealed a potential mechanical trigger for quiescent stem cells to activate healing processes. By probing the interaction of light with the molecular structure of periosteum [second harmonic and two photon imaging (Ng et al., 2017c)], submicron resolution maps of collagen and elastin fibers in the tissue were rendered. When applied to freshly excised bone and periosteum *in situ*, it was observed that the intrinsic crimp or curl of collagen fibrils relaxes when the periosteum is released from the underlying bone via cutting of the Sharpey’s fibers (Figure 1D). The relaxation in the curl was observed to coincide with rounding up of the resident stem cells adhering to the tissue fibers, providing a direct trigger for the cells to revert from quiescent to active states, and to initiate the genesis of new tissue templates associated with postnatal healing and prenatal development (Ng et al., 2017c).

Excitingly, the release of the tissue’s intrinsic prestress had a significant impact on another property of the tissue, namely its permeability. Permeability is an essential functional boundary property (Figure 2E1) for periosteum, which serves as an interface between bone and surrounding muscle. Release of prestress in the tissue was associated with a significant reduction in permeability of periosteum. Furthermore, permeability of tissue-similar fluid (Ringer’s lactate) through the periosteum exhibited direction- and flow-rate dependence. Permeability of periosteum increased eight to 16× when the flow rate was increased 120×. Surprisingly, this effect was much more pronounced in the bone to muscle direction than in the muscle-to-bone direction, a characteristic of a non-linear hydraulic valve (Evans et al., 2013a,b,c).

### Bone

Further smart properties of bone reveal themselves when one analyzes the different phases of the tissue itself. During development *in utero* and during postnatal healing, bone starts as a soft template of collagen and elastin that mineralizes over time (Knothe Tate, 2011b; Ng et al., 2017c). The temporal aspect of bone mineralization and maturation can be tracked using fluorochromes of different excitation and emission wavelengths (Figure 1E; different spectra excite different fluorophores). The fluorochromes chelate chemically to the mineral when it is laid down onto (nucleates around the fibrils of) the bone template; intramuscular or subcutaneous injection of fluorochromes over the 4-month-healing cycle reveals the temporal dynamics of mineralization, e.g., of a tissue template formed via periosteum-derived stem cells ingressing into a bone defect (Figure 1E). Whereas initial mineralization forms a disorganized scaffold referred to as woven bone (red, Figure 1E), subsequent tissue genesis and mineralization occurs layer by layer, typically in proximity to the vascular supply (green and then turquoise, in time intervals comprising weeks, Figure 1E) (Knothe Tate et al., 2011b).

Intrigued by the observation of counterintuitive flows in experimentally based computational models of bones, we aimed to test the hypothesis that non-homogeneous distributions of different caliber pores in bone would result in such counterintuitive flows, e.g., imbibement of fluid under compression. Using high resolution microscopy, bone’s different caliber porosities (pericellular versus vascular pores) were rendered as heat maps with warm colors depicting areas of high density and cool colors showing areas of low density (Figure 2D; Knothe Tate, 2003; Steck et al., 2003; Steck and Knothe Tate, 2005; Knothe Tate et al., 2009; Sidler et al., 2018). We then re-ran our computational models, which showed that specific patterns of porosity of different calibers indeed confer emergent, flow-directing properties to the tissue under mechanical loads (Knothe Tate et al., 2011b).

## Microscopy-Aided Design and Manufacture (Madame) of Bio-Inspired Materials and Structures

### Concept – Recursive Logic to Emulate Natural Tissues

Once we were able to quantify and precisely describe, quantitatively in four dimensions (4D), multiscale structure and emergent functional properties of natural materials, we then aimed to develop methods to design and manufacture new materials emulating nature’s own. This resulted in the development of a novel process, referred to as MADAME, to map spatial and temporal properties of smart, natural materials (Figures 2F,G; Knothe Tate, 2017b; Sidler et al., 2018). The process uses imaging and advanced computational methods to visualize patterns intrinsic to the material (Figures 1, 2). Using recursive logic, the basis of computer algorithms, these patterns are recreated digitally using computer-aided design principles, and physically using computer-assisted weaving and/or knitting, alone and/or in combination with multidimensional additive manufacturing, e.g., 3D printing, stereolithography and laser sintering (Reynolds, 1883; McBride et al., 2008; Knothe Tate, 2016, 2017b; Yu et al., 2017).

The Jacquard loom was the earliest computer – in 1801, a century prior to “the first punch card driven computers, the Jacquard loom wove patterns using loops of paper with holes to guide when hooks fell through the paper loop (hook down) or stayed above the loop (hook up), thereby encoding binary patterns of e.g., tapestry weaves” (McBride et al., 2008; Song et al., 2013; Knothe Tate, 2016, 2017a; Sidler et al., 2018). The computer-controlled Jacquard looms and additive manufacturing systems enable creation of physical embodiments (textiles, composites) of mechanical and other biophysical and spatiotemporal patterns intrinsically encoded in natural materials.

**FIGURE 3:**
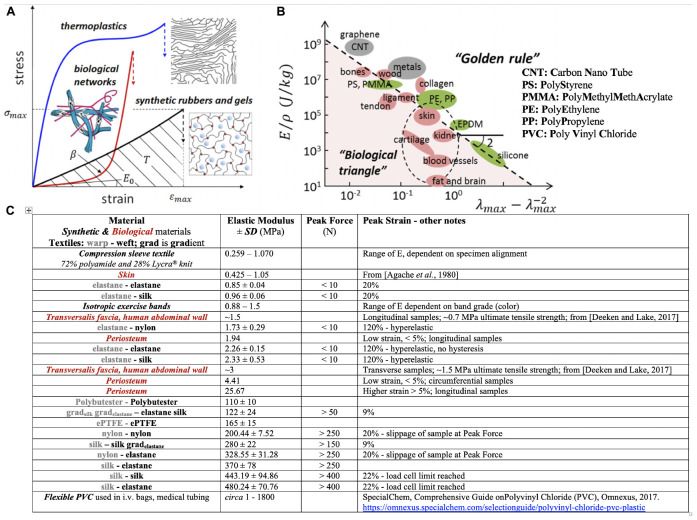
Sheiko and Dobrynin’s concept of encoding mechanical phenotype in synthetic polymeric materials and Knothe Tate’s recursive logic approach to encoding mechanoactive material properties in textile (and composite) patterns comprising fibers of different mechanical phenotypes. **(A)** Thermoplastics, synthetic elastomers and gels and biological networks (extracellular matrix, tissue) exhibit mechanical phenotypes exemplified through the materials’ stress *σ*- strain ε curve (E_0_, the Young’s Modulus, is the material stiffness in the linear elastic region of the curve; β shows strain stiffening or non-linear increase in Modulus with increase in deformation, a measure of material firmness; *σ_*max*_* is the strength of the material or stress at break; the area under the curve indicates material toughness or how much energy is dissipated at breakage.**(B)** Elongation at break (λ_max_) versus Young’s modulus (E) over the mass density (ρ) of a material. “This representation recovers scaling relations E ∼λ_max_-2, for elastomeric materials with λ_max_ ≫ 1, and E ∼ε_max_-2, for hard materials with strain at break ε_max_ = λ_max_-1 ≪ 1.” Panels **(A,B)** *used with permission* from Sheiko and Dobrynin (2019) and Vatankhah-Varnosfaderani et al. (2017). **(C)** Master Table of properties for materials created using MADAME and tested in tension, compared to *textile* and *isotropic control materials*, *using data from* Ng et al. (2017a,b,c, 2019, 2020).

More recent (2019) approaches (Sheiko and Dobrynin, 2019) suggest application of recursive logic for polymer design and engineering to mimic a range of mechanical properties appropriate for biological applications (Figure 3). While reduction to practice is currently in homogenous materials without intrinsic anisotropy or mechanical gradient properties (Vatankhah-Varnosfaderani et al., 2017), the concept expands upon the idea of encoding material properties architecturally, at molecular length scales, to achieve a range of biologically relevant mechanical properties previously achievable only empirically, through mixing of “various polymers, solvents and fillers” (Sheiko and Dobrynin, 2019). In combination with the aforementioned MADAME approach, a range of architectures might be achievable that range from molecular to micro- to macro- and meso-length scales. In particular, molecular encoding of polymers may provide a means to tune fiber and matrix mechanical properties for composite, advanced manufactured materials and products (Knothe Tate, 2019). In the future, it may enable real time manufacture of gradients materials through adjustment of the molecular composition of e.g., 3D printers and other advanced manufacturing platform materials.

### Incorporation of Pre- and Residual Stresses in Mechanoactive Material Design and Manufacture

Once we implemented additive manufacturing with textile engineering to create composite structures, we aimed to integrate prestresses and/or residual stresses, found in natural materials, into advance-manufactured smart materials. A two-pronged solution proved most effective. First we used a pre-tensioning system with the Jacquard loom to selectively prestress warp and weft (orthogonal weave) fiber directions (Knothe Tate, 2017a; Knothe Tate and Thrasou, 2017). Then we incorporated principles of *Kirigami*, the Japanese art of folding and cutting paper into shapes, to cut and/or preform fibers and/or fiber composites with defined residual stresses and dimensions, from sheets or composites of desired materials (Knothe Tate, 2020). Stresses were thereby defined by the geometry of the cuts, e.g., spiral versus zig-zag with characteristic dimensions defining magnitudes of stresses and their gradients, or by the architecture of the composite fibers. While kirigami has been applied in electroactive materials (Cartolano et al., 2019), its use in mechanoactive materials is novel, in particular in combination with weaving, knitting and additive manufacturing applications. In developing these mechanisms and manufacturing strategies (below), the design thinking approach of empathizing with one’s cells is particularly useful to brainstorm for new design elements and methods of reducing them to practice.

### Manufacturing Considerations

Application of design thinking approaches that use the visualization tool of empathizing with cells that manufacture tissues, stimulates conceptualization of new manufacturing processes and/or pipelines. When one observes a range of natural architectures across phyla of the animal and plant kingdoms, from bone to corals (animal kingdom), and wide-ranging plants, the aforementioned geometric and/or directional/anisotropic paradigms are reiterated again and again, and thereby create an infinite number of structures and associated mechanoactive functions. In summary, empathizing with cells and understanding their unique structural and functional capacities facilitates development of new manufacturing methods and processes at different length and time scales (Knothe Tate et al., 2009, 2016a; Knothe Tate, 2011a, 2017b; Ng et al., 2017c, 2019). While surface based growth of biological structures is rapidly emulatable in additive manufacturing, new manufacturing modalities need to be developed further for other architectures and anisotropic properties. These manufacturing pipelines may incorporate steps typically used in composite structure and sandwich structure design, with modular assembly or they may involve novel incorporation of mechanoactive fiber assembly into textiles which themselves are assembled within photopolymerizable resin matrix and/or sintering powders. In other words, multimodal advanced manufacturing will likely follow in the footsteps of multimodal imaging, according to the MADAME paradigm described above. In materials not incorporating living cells, clever designs can be invented to actively modulate force balances at interfaces, much in the ways that cells do in living materials (Sidler et al., 2018).

## Applications, Future Directions and Conclusion

Mechanoactive materials first found applications in tissue engineering for the development of tissue templates and scaffolds, as a direct translation from its inspiration (Anderson and Knothe Tate, 2007; Kuo and Tuan, 2008; Lujan et al., 2011; Song et al., 2013; Cartolano et al., 2019). In parallel, the mechanoactive materials were introduced for internal (implants) and external medical devices, mainly in cardiovascular and orthopedic fields of use (Knothe Tate et al., 2011a; Ng et al., 2020). New classes of mechanoactive materials will find uses *i.a.* in the medical and health sectors (Ng et al., 2017a; Hageman et al., 2018), in addition to safety and transport, military (Knothe Tate, 2011a), sports, and leisure wear sectors, cementing their disruptive status in the field of material science and advanced manufacturing (Knothe Tate et al., 2009; Sidler et al., 2018).

While biomimicry has inspired artists, scientists and engineers for well over five centuries (*cf.* da Vinci’s flying machine), the advent of advanced imaging has provided a critical enabling technology to decipher mechanisms underpinning emergent properties of natural materials and systems at multi-length and -time scales (Knothe Tate, 2017a,b). Such advances enabled through imaging have forged a path for translation of knowledge via design and manufacture of materials emulating the emergent properties of nature’s own (Knothe Tate, 2017b; Sidler et al., 2018). Such properties integrate sensor, transducer and actuator functions in the material itself (Knothe Tate et al., 2016b; Sidler et al., 2018).

Microscopy-Aided Design And ManufacturE lends itself well for the deciphering, design and manufacture of emergent properties from nature (Sidler et al., 2018; Ng et al., 2019, 2020). This is expected to result in parallel advances in innovative technological solutions and manufacturing of new materials and products harnessing those solutions. Visualization through high resolution spatiotemporal imaging is particularly enabling for mechanoactive materials, as the field of mechanics has a rich history of translating visualization of mechanical properties to elucidation of mechanisms and new inventions.

Similitude theory implements parametric scaling to study mechanics in very large- or small-scale systems where direct measures were not possible with then state-of-the-art technology (da Vinci, 1508; Einstein, 1911; Bridgman, 1922; Astarita, 1997; Anderson et al., 2008). For example, in aeronautics, an airplane or wing was typically scaled down in size to a model system on which flow parameters could be measured experimentally which would be impossible on the true-to-scale system. The scaling relationship enables linking of the model parameters to those of the actual system. In fluid dynamics, “qualitative and quantitative descriptions of flow regimes can be obtained using scaling relationships if viscous and inertial forces (Reynolds number) are maintained across the length scales (Reynolds, 1883, 1895; Rayleigh, 1892, 1904, 1915; Anderson et al., 2008; Sidler et al., 2018; Sheiko and Dobrynin, 2019).

In combination with MADAME, similitude theory may lead to new fundamental discoveries as well as design approaches and manufacturing methods in previously intractable nano-micro biological systems, enabling visualization of biophysical phenomena below the traditional realm of visualization (Anderson et al., 2008). In this way, MADAME serves as an enabling technology that, in combination with century-old theories such as similitude, may open the path toward new discoveries and inventions while also providing a path to manufacture materials and products with bio-inspired properties and mechanisms.

## Disclosure

MK has intellectual property patented and pending patent around multilayered surgical membranes as well as the design and manufacture of advanced manufactured composites that emulate nature’s own. The commercialization of these technologies is at an early (pre-revenues generating) stage. This manuscript reports scientific outcomes designed to benefit the field as a whole and does not report on any particular product or prototype with potential commercial interest.

## Ethics Statement

The animal study was reviewed and approved by the Commission for the Ethical Use and Care of Animals, Grisons, Switzerland.

## Author Contributions

MK wrote the perspective article which reflects her perspectives on the field.

## Conflict of Interest

MK is the inventor of several patented and patent pending technologies (Knothe Tate, 2016, 2018, 2019) and has an interest in a start-up company that was formed to commercialize these technologies. The pre-revenues stage start-up company is focused on R&D, and the current manuscript reflects the scientific findings of this R&D work in addition to the MK’s university-based laboratory endeavors.

## Footnotes

+ In contrast to undifferentiated cells, highly specialized, terminally differentiated cells exert low potential to become different specialized cell types, a process that can theoretically, albeit rarely, occur through dedifferentiation and redifferentiation, or cross-differentiation.

## Abbreviations

EMT: Epithelial to Mesenchymal Transition
MADAME: Microscopy-Aided Design And ManufacturE
MET: Mesenchymal to Epithelial Transition
Multi-D: Multi-Dimensional.
